# *IDH1* as a Cooperating Mutation in AML Arising in the Context of Shwachman-Diamond Syndrome

**DOI:** 10.3389/fonc.2019.00772

**Published:** 2019-08-14

**Authors:** Stéphanie Mourad, Mélanie Bilodeau, Mathieu Roussy, Louise Laramée, Luc Boulianne, Alexandre Rouette, Loubna Jouan, Patrick Gendron, Michel Duval, Pierre Teira, Josée Hébert, Henrique Bittencourt, Yves Pastore, Josette-Renée Landry, Sonia Cellot

**Affiliations:** ^1^Pediatric Hematology-Oncology Division, Charles-Bruneau Cancer Center, Centre Hospitalier Universitaire Sainte-Justine, Montreal, QC, Canada; ^2^Division of Hematology-Oncology, Montreal Children's Hospital, McGill University, Montreal, QC, Canada; ^3^Faculty of Medicine, Université de Montréal, Montreal, QC, Canada; ^4^Department of Biomedical Sciences, Université de Montréal, Montreal, QC, Canada; ^5^Department of Pathology, McGill University, Montreal, QC, Canada; ^6^Integrated Centre for Pediatric Clinical Genomics, CHU Sainte-Justine, Montreal, QC, Canada; ^7^Bioinformatics Core Facility, Institute for Research in Immunology and Cancer, Université de Montréal, Montreal, QC, Canada; ^8^Division of Hematology, Maisonneuve-Rosemont Hospital, Montreal, QC, Canada; ^9^Quebec Leukemia Cell Bank, Maisonneuve-Rosemont Hospital, Montreal, QC, Canada; ^10^Institute for Research in Immunology and Cancer, Université de Montréal, Montreal, QC, Canada; ^11^Streamline Genomics, Montreal, QC, Canada

**Keywords:** Shwachman-Diamond syndrome, predisposition syndrome, pediatric patient, leukemia, molecular profiling, cooperating mutation

## Abstract

Shwachman-Diamond syndrome (SDS) is a rare and systemic disease mostly caused by mutations in the *SBDS* gene and characterized by pancreatic insufficiency, skeletal abnormalities, and a bone marrow dysfunction. In addition, SDS patients are predisposed to develop myelodysplastic syndromes (MDS) and acute myeloid leukemia (AML), typically during adulthood and associated with *TP53* mutations. Although most SDS diagnoses are established in childhood, the nature and frequency of serial bone marrow cell investigations during the patients' lifetime remain a debatable topic. The precise molecular mechanisms leading to AML progression in SDS patients have not been fully elucidated because the patient cohorts are small and most disease monitoring is conducted using standard histological and cytogenetic approaches. Here we report a rare case of a patient with SDS who was diagnosed with AML at 5 years of age and survived. Intermittent neutropenia preceded the AML diagnostic but serial bone marrow monitoring according to the standard of care revealed no cytogenetic anomalies nor signs of clonal hematopoiesis. Using next generation sequencing approaches to find cytogenetically cryptic pathogenic mutations, we identified the cancer hotspot mutation c.394C>T/p.Arg132Cys in *IDH1* with high variant allelic frequency in bone marrow cells, suggesting clonal expansion of a major leukemic clone karyotypically normal, in the SDS-associated AML. The mutation was somatic and likely occurred at the leukemic transformation stage, as it was not detected in a matched normal tissue nor in bone marrow smear prior to AML diagnosis. Gain-of-function mutations in *IDH1*, such as c.394C>T/p.Arg132Cys, create a neo-activity of isocitrate dehydrogenase 1 converting α-ketoglutarate into the oncometabolite D-2-hydroxyglutarate, inhibiting α-ketoglutarate-dependent enzymes, such as histone and DNA demethylases. Overall, our results suggest that along with previously described abnormalities such as *TP53* mutations or monosomy7, 7q-, which are all absent in this patient, additional mechanisms including *IDH1* mutations drive SDS-related AML and are likely associated with variable outcomes. Sensitive techniques complementary to standard cytogenetics, such as unbiased or targeted panel-based next generation sequencing approaches, warrant testing for monitoring of myelodysplasia, clonal hematopoiesis, and leukemia in the context SDS. Such analyses would also assist treatment decisions and allow to gain insight into the disease biology.

## Background

Shwachman-Diamond syndrome (SDS) is a rare autosomal recessive bone marrow failure syndrome characterized by exocrine pancreatic insufficiency, bone abnormalities, progressive cytopenia, and predispositions to myelodysplastic syndrome (MDS) and acute myeloid leukemia (AML). The risk of worsening cytopenia(s), clonal myeloid evolution or AML is estimated at 20% by age 18 (1). The Severe Chronic Neutropenia International Registry reported a 1% per year progression rate to MDS/AML in patients with SDS, with a cumulative risk of MDS/AML reaching 36% by 30 years of age (2).

As the median age at diagnosis of SDS-associated AML is reported to be between 18 and 20 years and as the risk of AML increases with age (3), there are very few pediatric cases reported in the literature (<10 to our knowledge in the last 25 years, Table 1). As such, the molecular events driving malignant transformation in SDS are poorly described, and recent evidence points to a leukemia promoting bone marrow niche in SDS (6). The scant reported pediatric cases of SDS-related AML in the literature are mostly characterized by patient age, cytogenetic profile, and outcome. To our knowledge, all pediatric reported cases of SDS-associated AML occurred between 2.5 and 13 years of age (2, 4, 5) and had cytogenetic abnormalities discernable on karyotype, with frequent chromosome seven involvement (2, 4, 5). Furthermore, these patients had exceedingly poor outcome with only one reported case of survival in a 13-year-old child with SDS following hematopoietic stem cell transplant for AML [(5); Table 1].

**Table 1:** Reported cases of pediatric SDS-related AML in the literature between 1994 and 2019.

**Reference**	**Case**	**Age at diagnosis of AML (year)**	**AML FAB classification**	**Cytogenetics**	**Outcome**
Donadieu et al. (2)	1	2.5	AML with MDS-related changes FAB M2	Monosomy 7 (no further details)	Death, no HSCT
	2	7.3	AML with MDS-related changes FAB M6	45,XY,del(5)(q?q?),add(9)(q?), +11,add(17)(p?), −20,+22	Death, HSCT complicated by viral infections
	3	6	AML with preceding MDS FAB M4	46,XY,add(7)(q31)[15]/46,XY [1].ish der(7)t(1;7)(q32;q31)	Death, no HSCT
Smith et al. (4)	4	11	AML with preceding MDS FAB M5	47,XY, 1,del(9)(q22)	Death
	5	4.5	AML with preceding MDS FAB M5	47, XY, der(7)t(4;7)(q31;q11),+21 [13]	Death
	6	8	AML with preceding MDS FAB M2	46,XY,add(11)(p?), −15, −22, +mar1, +mar2	Death
	7	6	AML with preceding MDS FAB M6	Not assessed	Death
Valli et al. (5)	8	13	AML with preceding MDS FAB NA	46,XY,der(6)t(3;6)(q26.3;q16.1)[13]	Alive, post HSCT
	9	9	AML with preceding MDS FAB NA	45,XX,der(7)t(7;18)(p11;?),-18 [7]/43,XX,der(5)t(5;6)(q?28;q?13),der(6)t(6;13)(q21;q?),del(6)(q?13), der(7)t(7;18)(p11;?),−13,der(15)t((del(15)(q22);22)(p11.2;p11.2),−18 [14]	Death

*SDS, Shwachman-Diamond; AML, acute myeloid leukemia; MDS, myelodysplastic syndrome; HSCT, hematopoietic stem cell transplant; FAB, French-American-British classification of AML; NA, not available; ?, questionable identification of a chromosome or chromosome structure*.

In addition to the paucity of pediatric cases of SDS-related AML, limited molecular profiling data are available. A recent report suggests that acquisition of *TP53* mutations are initiating events contributing to the development of MDS/AML in SDS and may confer a poor prognosis (7). However, no other alteration is known to have any prognostic value.

Here we report a unique case of SDS-associated AML in a young pediatric patient, in absence of cytogenetic abnormalities. Using next-generation sequencing approaches to identify cytogenetically cryptic pathogenic mutations, we reveal an acquired somatic mutation in the Isocitrate Dehydrogenase 1 gene (*IDH1*), which is a rare cooperating event in pediatric AML and has never been reported in conjunction with SDS. Gain-of-function mutations in Isocitrate Dehydrogenase 1, an enzyme involved in cell metabolism, are known to modify the epigenetic landscape and to promote cancer progression (8). We propose that besides *TP53* mutations, additional cytogenetically silent mechanisms drive SDS-related myelodysplastic syndrome or AML, such as *IDH1* mutations, and next generation sequencing approaches may benefit the hematological evaluation and risk classification of patients.

## Case Presentation

**Figure 1:**
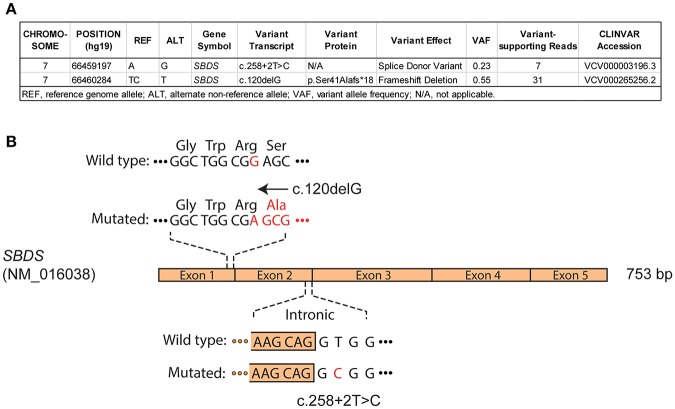
Molecular diagnosis of Shwachman-Diamond syndrome in a pediatric patient. Targeted sequencing of pre-leukemia blood DNA and **(A)** whole exome sequencing analysis of buccal swab DNA at AML diagnosis reveal compound heterozygous mutations impacting *SBDS* gene, consistent with an autosomal recessive Shwachman-Diamond syndrome. **(B)** Schematic representation of *SBDS* coding sequence (NM_016038), with confirmed c.120delG (exonic), and c.258+2T>C (intronic/splicing) mutations.

**Figure 2:**
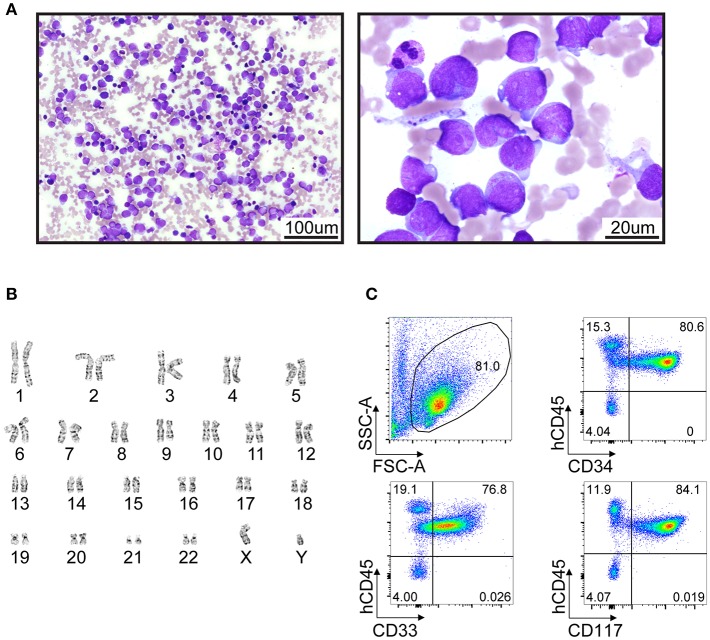
Clinical presentation of AML in a 5-year-old patient with SDS syndrome. **(A)** Wright**-**Giemsa-stained cytologic preparation showing leukemic blasts collected from a bone marrow aspiration. Imaging was performed with an upright microscope (Zeiss AxioScope A.1) equipped with 20X/0.5 plan-neofluar and 100x/1.4 oil objectives and an Axiocam 105 color camera. Left and right panels, 200X and 1000X magnification, respectively. **(B)** Standard GTG-banded karyotype revealing no cytogenetic abnormality. **(C)** Flow cytometry analyses of bone marrow cells suggest a myeloid leukemia (CD45^+^ CD33^+^ CD34^+^CD117^+^) with high infiltration (>80%).

A male infant was investigated at 7 months of life with failure to thrive secondary to exocrine pancreatic insufficiency, hypotonia, musculoskeletal congenital anomalies (syndactyly of toes, abnormally short ribs) and intermittent neutropenia with an absolute neutrophil count fluctuating between 0.8 ×10^9^/L and 4.2 ×10^9^/L, suggesting a diagnosis of SDS. The patient had an unremarkable medical family history and was referred to genetic counseling at the age of 1 year. The genetic basis of SDS was confirmed by sequencing of blood/buccal swab-derived DNA revealing two compound heterozygous mutations of the *SBDS* gene (intron 2 c.258+2T>C and exon 1 c.120delG (p.Ser41fs) (Figures 1A,B). He was followed with serial bone marrow aspirations, biopsies, and cytogenetic analysis, until the age of 5.5 years when he developed worsening cytopenia and his bone marrow evaluation revealed infiltration by 47.4% myeloblasts with underlying multilineage dysplasia (Figure 2A). These changes were compatible with a diagnosis of AML (M0, per FAB classification), with blasts expressing CD45^dim^, CD34^+^, CD33^+^, CD117+, DR^+^, MPO^+^, CD15^+^, CD13^partial^ on flow cytometry and with a normal karyotype (46,XY) on cytogenetic analysis (Figures 2B,C).

He was treated with a first cycle of induction chemotherapy (cytarabine, daunorubicin, etoposide), but minimal residual disease (MRD) remained positive at 0.4%, as assessed by flow cytometry at the end of the cycle. He pursued treatment with a second cycle consisting of high-dose cytarabine and etoposide, after which he achieved MRD negativity and proceeded to hematopoietic stem cell transplant. A first attempt with intra-osseous cord blood transplant following busulfan, fludarabine, and anti-thymocyte globulin (ATG) was unsuccessful with persistent aplasia. A second attempt with an alternate cord blood unit also failed to engraft after conditioning with total body irradiation, cyclophosphamide and fludarabine. Finally, the patient underwent a matched unrelated donor bone marrow transplant following conditioning with cyclophosphamide, fludarabine, and ATG. Hematopoietic engraftment was successful and the patient remains in first complete remission 5.5 years post-transplant (11-year-old).

**Figure 3:**
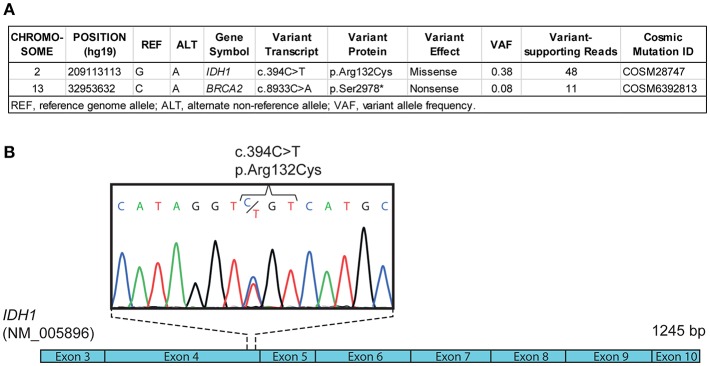
Molecular profiling of SDS-associated leukemia. **(A)** Differential whole exome sequencing analyses of leukemic bone marrow cell DNA compared with normal control (e.g., buccal swab DNA) reveal a somatic mutation (c.394C>T) in *IDH1* coding sequence, corresponding to the recurrent p.Arg132Cys mutation reported in adult AML. **(B)** Schematic representation of *IDH1* coding sequence (NM_005896) and Sanger sequencing chromatogram confirming the c.394C>T (p.Arg132Cys) mutation in leukemic bone marrow cell DNA.

Given his young age at diagnosis of SDS-associated AML and the lack of clonal cytogenetic anomalies, we searched for discrete oncogenic alterations such as indels and point mutations. First, array-based comparative genomic hybridization (aCGH) analysis did not reveal chromosomal copy-number abnormalities (data not shown) in leukemic cells, consistent with a normal karyotype. Then, whole exome and transcriptome sequencing were performed using bone marrow cells at diagnosis. A point mutation in *IDH1* (c.394C>T/p.Arg132Cys), reported to be pathogenic and recurrent in adult AML and other cancer types (8), was identified both by exome and transcriptome sequencing, with both wild-type and mutant alleles expressed at 45 and 55%, respectively (Figure 3A, Figure S1 and data not shown). The mutation was confirmed by Sanger sequencing (Figure 3B). Whole exome sequencing of buccal swab DNA confirmed that the *IDH1* mutation was somatic in origin as it was not found in a matched normal tissue (Figure S1). Somatic mutation of *IDH1* defined a major leukemic clone (variant allele fraction of 0.38 for a specimen with ~80% leukemic infiltration) while a minor clone with a mutation in *BRCA2* (variant allele fraction of 0.08) was also detected (Figure 3A). No fusion transcripts were identified from RNA sequencing reads using two different fusion detection tools (data not shown). The *TP53* gene was devoid of somatic alterations according to whole exome sequencing analysis (420X and 70X average coverage for leukemic bone marrow sample and buccal swab DNA, respectively, not shown). To estimate the timing of *IDH* mutation appearance, we performed NGS on DNA material obtained from scraping fixed bone-marrow smears, archived 6 months prior to AML diagnosis. *IDH1* c.394C>T (p.Arg132Cys) mutation was not detected in any of the 44 sequencing reads obtained spanning that position, indicating that the mutation was not present or below our detection threshold 6 months prior to AML diagnosis (Figure S1). These results suggest a cooperative role of *IDH1* somatic mutation in SDS-related AML progression, and warrant further mechanistic and multicenter clinical studies. Overall, these molecular analyses suggest that next-generation sequencing may reveal clonal evolution in cytogenetically normal SDS-associated myelodysplasia/leukemia as well as cooperating mutations.

## Discussion

SDS is a ribosomopathy characterized by genomic instability contributing to the occurrence of chromosomal abnormalities and therefore to the risk of malignant transformation to MDS/AML. Characterization of high-risk clonal chromosomal abnormalities remains a challenge, as some patients only develop transient genetic alterations devoid of malignant potential. The most commonly encountered chromosomal anomalies in SDS comprise isochromosome 7 (i(7)(q10)), resulting in a gain of long arm of chromosome 7 (7q), and deletion 20q (del(20)(q)) (5), not typically associated with initiating events in leukemogenesis. Deletion 20q has not yet been reported to have malignant transformation potential, unless associated with other cytogenetic changes (9). Overall, the risk of MDS/AML in patients with SDS increases with age and varies considerably according to the acquired cytogenetic abnormalities, with complex karyotypes seemingly conferring an increased risk of malignant transformation (2).

SDS being a rare disorder, thorough understanding of SDS-related AML has remained limited due to the small number of patients, especially in pediatrics. Given the heterogeneity of MDS/AML progression in patients with SDS, we postulated that acquired cooperating somatic mutations must contribute to malignant transformation. This should be especially true for pediatric patients in whom the risk of SDS-related MDS/AML is considerably lower than in adults, prompting our investigations for this case. The unique presentation of an SDS-associated AML in a young pediatric patient lacking chromosomal rearrangements and any pathogenic *TP53* mutation confirmed this hypothesis and revealed a cooperating genetic mutation in the Isocitrate Dehydrogenase 1 gene (*IDH1*), associated with promoting leukemia.

IDH1 encodes a key cytoplasmic and peroxisomal enzyme of the Krebs cycle responsible for the catalyzation of isocitrate to α-ketoglutarate while reducing NADP to NADPH and liberating CO_2_ (8). The mutant IDH1 enzyme acquires a novel molecular function by which it converts α-ketoglutarate to the oncometabolite D-2-hydroxyglutarate (8). D-2-hydroxyglutarate inhibits the α-ketoglutarate-dependent enzymes that regulate epigenetic modeling, collagen synthesis and cell signaling (10). *IDH1* mutations have been found to be rare in pediatric AML (~1–2%) (11–13), but to be present at a higher frequency in pediatric AML with a normal karyotype (~5%) (11). They have also been reported at the leukemic transformation of myeloproliferative diseases (14, 15) and, combined with *IDH2* mutations, to be present in ~5% of MDS patients (16). Somatic mosaic mutations of *IDH1* have been detected in hematologic malignancies (BCP-ALL and AML) of children with Maffucci syndrome, a non-hereditary disorder characterized by multiple enchondromas and hemangiomas (17, 18). In adult, detection of somatic mutations in *IDH1* in peripheral blood DNA is highly predictive of a future leukemia development (19).

To the best of our knowledge, we present the first case of oncogenic *IDH1* mutation identified in SDS-associated AML. Pediatric AML associated with *IDH1* mutations present a favorable overall survival (13) compared with adult AML with *TP53* alterations (20), but large multicentric cohort of patients will be necessary to better stratify the genetic subsets and outcomes of SDS-associated leukemia. This study reports one of the only cases of pediatric SDS-related AMLs who achieved complete remission and survived, raising the interesting hypothesis that it is ultimately the genetic makeup of the leukemia that impacts prognosis in the context of SDS, and not the underlying cancer predisposition syndrome in itself.

## Conclusions

The discovery of a cooperating *IDH1* mutation in SDS-associated AML illustrates the necessity to refine surveillance for malignant transformation in this condition. Standard cytogenetic analyses may fail to detect single nucleotide genetic alterations driving clonal hematopoiesis and leukemic progression. Additional sensitive techniques, such as unbiased or targeted panel-based next generation sequencing approaches, would benefit the monitoring of myelodysplasia, clonal hematopoiesis, and leukemia in the context SDS. The genetic landscape of SDS-related AML should be evaluated to establish the disease-risk stratification, to assist treatment decisions and ultimately to gain insight into the biology of a rare malignancy.

## Data Availability

This manuscript contains previously unpublished data. The name of the repository and accession number(s) are not available at the time of publication but will be available upon request.

## Ethics Statement

The Institutional Review Board of CHU Sainte-Justine approved the study and informed consent was obtained from the parents.

## Author Contributions

SM, J-RL, MB, AR, and SC wrote the manuscript. SM, J-RL, MB, MR, LL, and AR generated the figures and table. SM, J-RL, MB, MR, LL, LB, AR, LJ, and PG contributed to experiments and analyses. YP, MD, PT, HB, and SC were in charge of the patient. JH and SC supervised the biobanking resource. All authors revised and approved the manuscript.

### Conflict of Interest Statement

J-RL is a founder and has ownership stake in Streamline Genomics. The remaining authors declare that the research was conducted in the absence of any commercial or financial relationships that could be construed as a potential conflict of interest.

## References

[1] Hashmi SK Allen C Klaassen R Fernandez CV Yanofsky R Shereck E. Comparative analysis of Shwachman-Diamond syndrome to other inherited bone marrow failure syndromes and genotype-phenotype correlation. Clin Genet. (2011) 79:448–58. 10.1111/j.1399-0004.2010.01468.x 20569259

[2] Donadieu J Fenneteau O Beaupain B Beaufils S Bellanger F Mahlaoui N. Classification of and risk factors for hematologic complications in a French national cohort of 102 patients with Shwachman-Diamond syndrome. Haematologica. (2012) 97:1312–9. 10.3324/haematol.2011.057489 22491737 PMC3436231

[3] Myers KC Davies SM Shimamura A. Clinical and molecular pathophysiology of Shwachman-Diamond syndrome: an update. Hematol Oncol Clin North Am. (2013) 27:117–28. 10.1016/j.hoc.2012.10.003 23351992 PMC5693339

[4] Smith OP Hann IM Chessells JM Reeves BR Milla P. Haematological abnormalities in Shwachman-Diamond syndrome. Br J Haematol. (1996) 94:279–84. 10.1046/j.1365-2141.1996.d01-1788.x 8759887

[5] Valli R De Paoli E Nacci L Frattini A Pasquali F Maserati E. Novel recurrent chromosome anomalies in Shwachman-Diamond syndrome. Pediatr Blood Cancer. (2017) 64:e26454. 10.1002/pbc.26454 28130858

[6] Zambetti NA Ping Z Chen S Kenswil KJG Mylona MA Sanders MA. Mesenchymal inflammation drives genotoxic stress in hematopoietic stem cells and predicts disease evolution in human pre-leukemia. Cell Stem Cell. (2016) 19:613–27. 10.1016/j.stem.2016.08.021 27666011

[7] Xia J Miller CA Baty J Ramesh A Jotte MRM Fulton RS. Somatic mutations and clonal hematopoiesis in congenital neutropenia. Blood. (2018) 131:408–16. 10.1182/blood-2017-08-801985 29092827 PMC5790127

[8] Raineri S Mellor J. IDH1: linking metabolism and epigenetics. Front Genet. (2018) 9:493. 10.3389/fgene.2018.00493 30405699 PMC6206167

[9] Maserati E Pressato B Valli R Minelli A Sainati L Patitucci F. The route to development of myelodysplastic syndrome/acute myeloid leukaemia in Shwachman-Diamond syndrome: the role of ageing, karyotype instability, and acquired chromosome anomalies. Br J Haematol. (2009) 145:190–7. 10.1111/j.1365-2141.2009.07611.x 19222471

[10] Cairns RA Mak TW. Oncogenic isocitrate dehydrogenase mutations: mechanisms, models, and clinical opportunities. Cancer Discov. (2013) 3:730–41. 10.1158/2159-8290.CD-13-0083 23796461

[11] Andersson AK Miller DW Lynch JA Lemoff AS Cai Z Pounds SB. IDH1 and IDH2 mutations in pediatric acute leukemia. Leukemia. (2011) 25:1570–7. 10.1038/leu.2011.133 21647154 PMC3883450

[12] Bolouri H Farrar JE Triche T Jr Ries RE Lim EL Alonzo TA The molecular landscape of pediatric acute myeloid leukemia reveals recurrent structural alterations and age-specific mutational interactions. Nat Med. (2018) 24:103–12. 10.1038/nm.4439 29227476 PMC5907936

[13] Damm F Thol F Hollink I Zimmermann M Reinhardt K van den Heuvel-Eibrink MM. Prevalence and prognostic value of IDH1 and IDH2 mutations in childhood AML: a study of the AML-BFM and DCOG study groups. Leukemia. (2011) 25:1704–10. 10.1038/leu.2011.142 21647152

[14] Tefferi A Lasho TL Abdel-Wahab O Guglielmelli P Patel J Caramazza D IDH1 and IDH2 mutation studies in 1473 patients with chronic-, fibrotic- or blast-phase essential thrombocythemia, polycythemia vera or myelofibrosis. Leukemia. (2010) 24:1302–9. 10.1038/leu.2010.113 20508616 PMC3035975

[15] Green A Beer P. Somatic mutations of IDH1 and IDH2 in the leukemic transformation of myeloproliferative neoplasms. N Engl J Med. (2010) 362:369–70. 10.1056/NEJMc0910063 20107228

[16] Sperling AS Gibson CJ Ebert BL. The genetics of myelodysplastic syndrome: from clonal haematopoiesis to secondary leukaemia. Nat Rev Cancer. (2017) 17:5–19. 10.1038/nrc.2016.112 27834397 PMC5470392

[17] Akiyama M Yamaoka M Mikami-Terao Y Ohyama W Yokoi K Arakawa Y. Somatic mosaic mutations of IDH1 and NPM1 associated with cup-like acute myeloid leukemia in a patient with Maffucci syndrome. Int J Hematol. (2015) 102:723–8. 10.1007/s12185-015-1892-z 26508204

[18] Hirabayashi S Seki M Hasegawa D Kato M Hyakuna N Shuo T. Constitutional abnormalities of IDH1 combined with secondary mutations predispose a patient with Maffucci syndrome to acute lymphoblastic leukemia. Pediatr Blood Cancer. (2017) 64:e26647. 10.1002/pbc.26647 28544751

[19] Desai P Mencia-Trinchant N Savenkov O Simon MS Cheang G Lee S. Somatic mutations precede acute myeloid leukemia years before diagnosis. Nat Med. (2018) 24:1015–23. 10.1038/s41591-018-0081-z 29988143 PMC6849383

[20] Hou HA Chou WC Kuo YY Liu CY Lin LI Tseng MH. TP53 mutations in *de novo* acute myeloid leukemia patients: longitudinal follow-ups show the mutation is stable during disease evolution. Blood Cancer J. (2015) 5:e331. 10.1038/bcj.2015.59 26230955 PMC4526785

