# Supplementary material for: IDH1 as a Cooperating Mutation in AML Arising in the Context of Shwachman-Diamond Syndrome
Source: Front Oncol. 2019 Aug 14;9:772. doi: 10.3389/fonc.2019.00772 (PMC6702516; doi:10.3389/fonc.2019.00772)
Supplement: Supplementary file 1 [file Image_1.pdf]

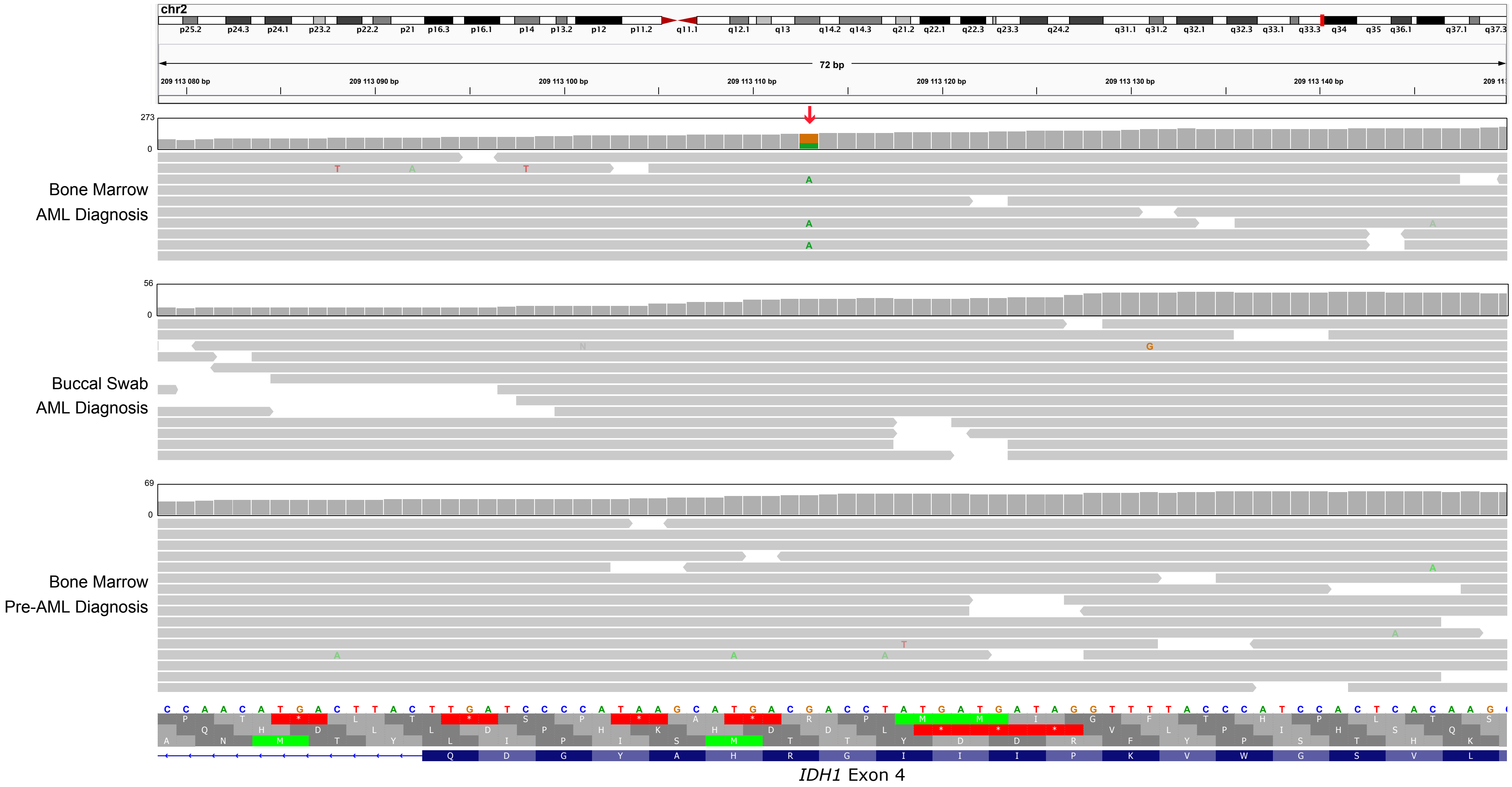

Figure S1. Somatic *IDH1* mutation identified in Shwachman-Diamond syndrome-associated AML. Aligned exome sequencing reads are shown for exon 4 of *IDH1* using Integrative Genomics Viewer for the indicated samples, with coverage on top graph. Samples, from top to bottom: DNA extracted from fresh tumor bone marrow at AML diagnosis, buccal swab DNA at AML diagnosis, and DNA extracted from fixed bone marrow smear 6 months pre-AML diagnosis. Mutation in *IDH1* is indicated by a red arrow.
